# Editorial: Occupational Neuroscience: Nervous System's Health at the Workplace

**DOI:** 10.3389/fnhum.2022.830288

**Published:** 2022-02-03

**Authors:** Sergio Garbarino, Nicola Magnavita, Franca Barbic, Erik Valdemar Stålberg, Paola Lanteri

**Affiliations:** ^1^Department of Neuroscience, Rehabilitation, Ophthalmology, Genetics, and Maternal/Child Sciences (DINOGMI), University of Genoa, Genoa, Italy; ^2^Postgraduate School of Occupational Medicine, Università Cattolica del Sacro Cuore, Rome, Italy; ^3^Department of Woman/Child and Public Health, Fondazione Policlinico Universitario Agostino Gemelli IRCCS, Rome, Italy; ^4^Department of Biomedical Sciences, Humanitas University, Milan, Italy; ^5^Internal Medicine, IRCCS Humanitas Research Hospital, Milan, Italy; ^6^Department of Neuroscience, Uppsala University, Uppsala, Sweden; ^7^Neurophysiology Unit, Fondazione IRCCS Istituto Neurologico Carlo Besta, Milan, Italy

**Keywords:** neurological disorders and brain damage, neurotoxic environment, occupational medicine (MeSH), sleep-wake cycle, stress

It is widely known that physical, chemical, biological, and psychological risk factor exposures in the working environment may impact the health, wellbeing, and safety of employees by producing acute or chronic alterations in complex functions controlled by the nervous system. At the same time, exposure to different multiple risk factors, potentially involving neural regulation systems in humans is increasing nowadays and requires a new complex multidisciplinary approach to the Research Topic (Thompson et al., 2020). Physical and psychological stress may enhance the neurodegenerative processes whose mechanisms are still poorly understood (Pearce and Kromhout, 2014). Occupational stress may seriously affect the autonomic nervous system as well as impacting cognitive performance and alertness. Stress causes include irregular and long working shifts that are often associated with physiologic wake-sleep rhythms alterations (Magnavita and Garbarino, 2017).

Long-term exposure to risk factors could amplify the effects also impacting the cardiovascular system, particularly in aging workers.

The recent development of immuno-neurology, neuro-epidemiology, and neuro-genetics provided tools and methods to improve the current knowledge on the role of the workplace and environmental exposure in reducing health and wellbeing (Ijomone et al., 2020).

Multiple Sclerosis, some polyradiculopathies, and synucleinopathies such as Alzheimer's and Parkinsonisms are thought to be tightly connected to environmental factors acting on an already genetically susceptible host. Genetic polymorphism studies and an in-depth investigation of the role of occupational and environmental exposure potentially translating into an effective epigenetic impact will help to identify specific populations of genetically susceptible individuals, for whom, different protection strategies should be adopted in the workplace (Hedström et al., 2016).

In addition to genetic polymorphism studies, electrophysiologic and neuroimaging studies, in particular, evoked potentials, electromyography, conduction velocities, nerve and muscle echography, and MRI, can help to confirm the etiopathogenesis, brain damage symptoms, to detect subclinical or early peripheral and central nervous system involvement and to accurately diagnose and manage the disease. This knowledge will help to further develop personalized occupational health, promptly identifying and protecting those workers who are resulting more susceptible to neurotoxic effects.

This Research Topic on “*Occupational Neuroscience: Nervous System's Health at the Workplace*” has aimed at stimulating scientists and clinicians to focus on the potential relationships between nervous system disorders, wellbeing alteration, and the complex occupational environment's impact on the nervous system's health, providing inputs and novel methodological approaches. This area seeks to analyze the effects in a holistic fashion, adopting a multidisciplinary approach by involving differently specialized experts.

Even though it is an innovative matter that it tried to join the neuroscience and the occupational world, most of manuscripts were submitted by specialists from neuroscience field.

Following an Occupational health traditional approach, Lucero and Muñoz-Quezada have analyzed the effects of occupational exposure to pyrethroid pesticides on the neurobehavioral and neurocognitive functioning of agricultural workers through a systematic review.

Benzodiazepines have been widely used in clinical practice for over four decades and continue to be one of the most consumed and highly prescribed class of drugs available in the treatment of anxiety, depression, and insomnia, for the first time in Literature, Garbarino et al. presented a systematic review and metanalysis about occupational injuries and use of benzodiazepines.

An abnormal quantity or quality of sleep, often due to shiftwork in the workplace, is associated with many diseases, and is also evidence that poor sleepers are more frequently absent from work, have more accidents, and are less productive. The possible damage caused by a reduction in the quantity or quality of sleep can be more serious in the caring professions as addressed by Alfonsi et al. The authors provided an updated overview of the literature on sleep problems in night shift nurses and critically analyzed the psychosocial factors defining an effective countermeasure based on an individualized approach.

In a variety of helping professions emotional stress can lead to “burnout.” Alsalhe et al. explored burnout syndrome among teachers, a human service professionals particularly vulnerable to occupational burnout since highly demanding and challenging task.

Severe orthostatic intolerance with recurrent syncope while standing are the two most disabling manifestations of Pure Autonomic Failure (PAF), a rare peripheral synucleinopathy affecting mild-age subjects (Kaufmann, 1996). Zamunèr et al. found significant correlations between Work Ability Index and cardiovascular autonomic control biomarkers in response to gravitational stimulus in a group of PAF patients still engaged in working activity. The results of the study open to new preventive strategies in workplace to delay early retirement and improve working performance of patients.

Health workers are central to the COVID-19 pandemic response, balancing additional service delivery needs while preserving access to essential health services and deploying COVID-19 vaccines. A case of recurrent COVID-19 disease with neurovestibular symptoms in a health worker is reported by Zaffina et al. The possibility of recurrence, reactivation or inflammatory rebound of SARS-CoV-2 infection was discussed in the light of Literature data.

Workplace participation of individuals with disabilities continues to be a challenge. An employee with neurological disorder should be providing all-round support to return to work. Brain tumors (BT) are between the eight most common cancers among persons aged 40 years, with an average survival time of 10 years for patients affected by non-malignant brain tumor. Some patients continue to work, reporting difficulties in work-related activities, or even job loss. Schiavolin et al. reviewed the existing information about the ability people with BT to return to work and to identify factors associated with job loss.

Silvaggi et al. investigated the differences about cognitive status, attention and executive functions, memory, word fluency, and functional status, emotional distress and disability between patients returning to work and those who did not after brain tumor surgery.

Although counts of occupational neurological disorder compose only a small part of the overall occupational disorders, it has a significant impact on the occupational safety and health system. With this Research Topic, we have promoted an increased attention and interest in strengthening the existing ties and dialogue between neuroscience and occupational health from prevention, to diagnosis and management up labor reintegration strategies of workers. Workplace participation of individuals with disabilities continues to be a challenge. From a broader perspective, this improvement might help to reduce the percentage of work leave due to disease, reduce workplace accidents, and improve the mental health of workers.

## Author Contributions

All authors listed have made a substantial, direct, and intellectual contribution to the work and approved it for publication.

## Conflict of Interest

The authors declare that the research was conducted in the absence of any commercial or financial relationships that could be construed as a potential conflict of interest.

## Publisher's Note

All claims expressed in this article are solely those of the authors and do not necessarily represent those of their affiliated organizations, or those of the publisher, the editors and the reviewers. Any product that may be evaluated in this article, or claim that may be made by its manufacturer, is not guaranteed or endorsed by the publisher.
